# Taxonomic diversity of green plants (Viridiplantae) in the Caatinga Phytogeographic Domain: a study supported by environmental licensing

**DOI:** 10.3897/BDJ.13.e159621

**Published:** 2025-09-15

**Authors:** Vinicius Messas Cotarelli, Edson Gomes de Moura-Júnior, Liliane Ferreira Lima, Andre Paviotti Fontana, Lorenna Campos Cruz, Renato Garcia Rodrigues, Daniel Salgado Pifano

**Affiliations:** 1 NEMA/UNIVASF, Petrolina, Brazil NEMA/UNIVASF Petrolina Brazil

**Keywords:** Caatinga Phytogeographic Domain (CPD), floristic composition, Seasonally Dry Tropical Forest, shortfalls in biodiversity

## Abstract

**Background:**

Limited access to taxonomic and biogeographical data on plants across large spatial scales poses challenges for biodiversity research. This study examined richness, taxonomic contributions and biogeographic insights derived from a floristic inventory encompassing 56,144 km² of the Caatinga Phytogeographic Domain (CPD), carried out within the framework of environmental licensing for a water infrastructure project (São Francisco River Transposition Project – PISF). Over a period of fifteen years, specimens from various plant groups (algae, angiosperms, bryophytes, ferns and lycophytes) were collected and taxonomic, ecological and biogeographic data were compiled for the species occurring in the study area (e.g. threat status, life form, geographic origin, endemism within the CPD), resulting in a checklist and a biodiversity dataset. The number of plant samples and species richness recorded in the study area was compared with previously available data. Rarefaction curves were used to assess the increase in species richness in relation to sampling effort within the study area.

**New information:**

A total of 29,247 samples were collected, resulting in the identification of 1,610 species. Herbaceous plants represented the dominant group, accounting for 40.89% of the species richness. A total of 173 species endemic to the CPD were recorded, along with 16 species listed in the Brazilian national list of threatened species. Furthermore, 284 first occurrences of 110 species were registered for one or more states in the Northeast of Brazil or within the CPD. Two new species were described in collaboration with taxonomic specialists and the donation of duplicate samples to national herbaria led to the citation of five paratypes. The checklist increased the number of specimens previously recorded in the study area by 240% and expanded the known species richness by approximately 14%. The rarefaction curve computed from the dataset indicated a tendency towards stabilisation of species numbers in the area. These findings highlight the value of primary data generated through environmental licensing linked to infrastructure projects in addressing biodiversity knowledge gaps.

## Introduction

The complete understanding of any biodiversity feature (e.g. taxonomic diversity) in an ecosystem is practically unattainable due to nature's complex temporal and spatial dynamics and the limited human capacity to study it (Lomolino 2004). Spatial and temporal inequality in research efforts and infrastructure results in variations in the quality and reliability of available data for biodiversity studies and conservation planning, leading to knowledge shortfalls in biodiversity (Hortal et al. 2015). Two of these shortfalls point out knowledge gaps for Linnean taxonomy and Wallacean species distribution. The Linnean deficit refers to the discrepancy between the number of formally described species and the actual number of existing species, while the Wallacean shortfall arises from geographic biases in information about species distributions, making many maps of taxonomic diversity resemble maps of research effort (Lomolino 2004). These shortfalls impact our interpretation of macroecological phenomena and processes of biodiversity (Hortal et al. 2015), in addition to biodiversity conservation decision-making, such as selecting and valuing protected areas. In Brazil, the establishment of these areas in each Phytogeographic Domain is based on assessments of socio-environmental and macroecological characteristics of the territory (e.g. biogeography of endemic, rare and endangered species).

The first checklists of plants from the Caatinga Phytogeographic Domain - CPD (a territory of approximately 850,000 km^2^ in the Brazilian semi-arid region, comprising large areas of Seasonally Dry Tropical Forest and irregularly spaced patches of Tropical Forest, Savannah or Campo Rupestre, *sensu* Queiroz et al. (2017)) were produced in the last 10 years, based on compiled data from publications (Moro et al. 2014) or from herbarium and botanical collections available on digital platforms (Fernandes et al. 2020). Despite these publications having contributed to advancing the knowledge of plant diversity in the CPD, a portion of the taxonomic and biogeographic information of species in this Domain remains unknown due to lack of access to their habitats (Oliveira et al. 2016). It is estimated that less than 50% of the CPD territory has at least one plant collection or study with taxonomic and/or phytosociological objectives (Moro et al. 2014). In Brazil, field expeditions aimed at surveys of plant or animal species are predominantly supported by government funding agencies of scientific research, such as Conselho Nacional de Desenvolvimento Científico e Tecnológico (CNPq) and Fundação Coordenação de Aperfeiçoamento de Pessoal de Nível Superior (CAPES) (CNPq 2023, CAPES 2024). These expeditions tend to be spatially biased, occurring mainly near access roads, major cities and research centres (Oliveira et al. 2016, Carvalho et al. 2023).

Studies indicate that plant checklists prior to the installation of new infrastructure projects are important for expanding both taxonomic and biogeographic knowledge of species in the investigated territory and accessing the informations on the biological communities or ecosystems (Moura et al. 2018). Thus, in the recent context of diminishing financial resources for science in Brazil (Siqueira and Rocha 2017, Magalhães 2017), environmental actions linked to the licensing of infrastructure and human development projects can attend as viable strategies for accessing taxonomic diversity and obtaining data for biodiversity conservation. The Projeto de Integração do Rio São Francisco – PISF (in English, São Francisco River Transposition Project) is a work of infrastructure in Brazil that aims to integrate a perennial river (São Francisco River) with other hydrographic basins in north-eastern Brazil (ANA 2021). As a result, the PISF will provide water to more than 12 million people in the Brazilian semi-arid region who currently have limited access to this resource (ANA 2021). To meet the requirements of the installation licence for the PISF (Installation Licence No. 438/2007 - DILIC/IBAMA), researchers from the Universidade Federal do Vale do São Francisco - UNIVASF (in English, Federal University of São Francisco Valley) have been conducting botanical studies in the project's influence area with funding from the federal government (MIDR 2024). Amongst the research conducted, the floristic inventory stands out, aiming to access the taxonomic diversity of plants in the areas directly or indirectly affected by the PISF.

This study will present the results of the floristic surveys carried out in an area of approximately 56,000 km² of the CPD, accessed through actions linked to the PISF environmental licensing. Through this checklist, the secondary aims of the study were the following: i) to classify species based on life form, geographic origin, endemism and degree of extinction threat; ii) to present the contribution of this study to the taxonomic and biogeographic shortfalls of the flora in the CPD.

## Materials and methods

### Area and sampling period

**Figure 1.**
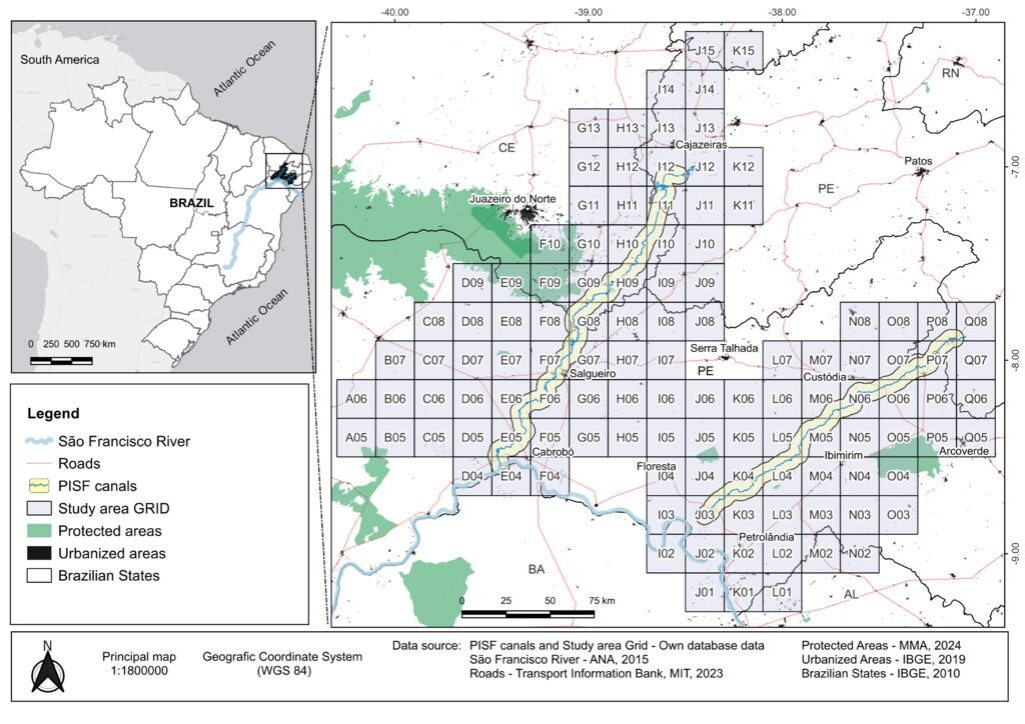
Location of the sampled area in Brazil with the limits of the sampling grid.

The study area is located within the Caatinga Phytogeographic Domain (CPD) between the meridians 40°30' W and 36°30' W and the parallels 6°10' S and 9°30' S in the semi-arid region of north-eastern Brazil. The delimitation is the area of direct influence of the PISF. To plan field expeditions over time, the study area was divided into a grid containing 116 grid cells of 22 × 22 km (approximately 484 km² each), totalling 56,144.00 km² (Fig. 1).

The study area encompasses five ecoregions of the CPD (ecoregions are land and water units delimited by biotic and abiotic factors that regulate the structure and function of the biological communities found within them, *sensu* Velloso et al. (2002)). They were: Depressão Sertaneja Meridional, Depressão Sertaneja Setentrional, Complexo do Araripe Ibipiaba, Raso da Catarina e Planalto da Borborema (in English, Southern Sertaneja Depression, Northern Sertaneja Depression, Ibipiaba-Araripe Complex, Raso da Catarina and Planalto da Borborema, respectively). The Northern and Southern Sertaneja Depression ecoregions represent 82.9% of the study area, Raso da Catarina represents 12.5%, Planalto da Borborema 3.1% and the Ibiapaba-Araripe Complex 1.5%.

Field expeditions were conducted between June 2008 and September 2023. Until 2011, wetlands or firm soil areas near the PISF construction canal were covered for the purpose of plant collection. From January 2012 onwards, class cells with low species richness and few plant samples were covered many times. Between years, field expeditions had different frequencies. Within the year, field expeditions in each class cell were conducted during the rainy season.

### Field collection, checklist, and species classification

Fertile plant specimens were collected, preserved and herborised following Judd et al. (2009). The collected specimens were georeferenced, obtaining geographic coordinates with the highest possible accuracy for the collection site. The geographic coordinates of these plant samples were interpolated with the grid cells of the study area.

While the focus was on accessing angiosperms, Green Macroalgae (hereafter referred to as Algae), Bryophytes (group including mosses, hornworts and liverworts) and Pteridophytes (group including ferns and lycophytes) were also collected. Botanists involved in this study performed the taxonomic identification of the plants, assisted by specialised bibliographies, photographs of exsiccate available in the SpeciesLink (2024), Flora e Funga do Brasil (2024) and Tropicos from the Missouri Botanical Garden (Tropicos 2024). Additionally, direct assistance was received from specialists in different taxonomic groups (genera, families or orders), who provided identifications after receiving duplicate specimens.

**Figure 2.**
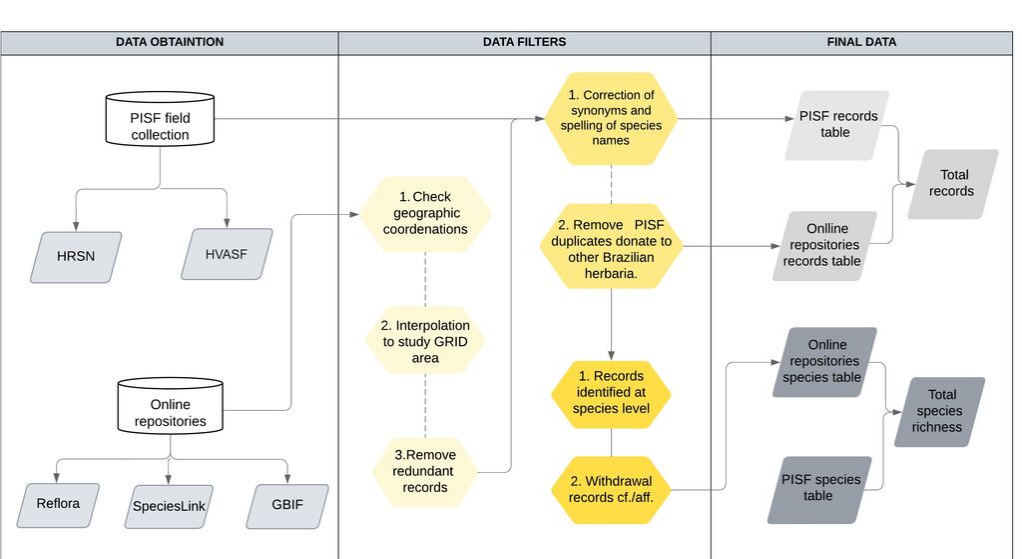
Flowchart containing the steps of data collection and screening.

For the construction of the checklist, specimens identified only at the genus or family level, as well as those requiring confirmation of the specific epithet as "cf." or "aff." [refer to Lucas (1986) for the application of these terms], were excluded (Fig. 2). Furthermore, the spelling of scientific names and author names for the species was verified and corrected as necessary, following the Flora e Funga do Brasil (2024) database. This was achieved using a routine in R software (R Core 2023), version 4.2, with the Flora R package, version 0.3.4 (Carvalho 2022). For species names not found in this database, additional sources were consulted, including Tropicos from the Missouri Botanical Garden Tropicos (2024), The Plant List (2024), AlgaeBase (Guiry and Guiry 2024) and the International Plant Names Index (IPNI 2024).

The taxonomic classification of species in the checklist followed Goffinet (2000) for Bryophytes, Lee (2012) for Algae, APG IV (2016) for angiosperms and PPG I (2016) for Pteridophytes. The Flora e Funga do Brasil (2024) was used to classify species in the checklist according to their origin (Naturalised non-native to Brazil; Cultivated non-native to Brazil; or Native to Brazil), endemism to CPD (Endemic or Non-endemic) and life form (Aquatic-benthos, Herb, Liana/climber/twiner, Palm, Subshrub, Thallose or Tree). For extinction threat criteria, species were classified, based on Ordinance 300/2022 of the Brazilian Ministry of the Environment – MMA (MMA 2022).

### Contribution to taxonomic and biogeographic shortfalls

To determine species occurrences in the States of north-eastern Brazil, the Flora e Funga do Brasil (2024) database was consulted. This procedure allowed us to assess whether the species in the checklist had new occurrences within these states. Additionally, it was verified whether species in the checklist were included in the current CPD plant list, as reported by Fernandes et al. (2020).

To address biodiversity shortfalls assessed using the PISF database, this dataset was compared with those available in online repositories containing herbarium and botanical collection data (SpeciesLink, Reflora and GBIF). Green plant samples with geographic coordinates from these repositories were selected and subsequently interpolated with the grid cells of the study area. Duplicate records, including those with identical herbarium vouchers or collector numbers, were eliminated. Furthermore, samples collected through the PISF project were excluded from the repository samples (Fig. 2). This process resulted in a dataset of green plants (samples and species) collected within the grid cells of the study area, derived either from online repositories or the PISF dataset (Fig. 2).

A data matrix of the samples number per species was created using field data from PISF and data from online repositories. This data matrix was used to generate rarefaction curves, which assess the trend of cumulative species numbers as a function of the increasing number of samples in the study area. These analyses were performed using data from online repositories, PISF field expeditions or a combination of both datasets. The iNEXT package version 3.0.0 (Hsieh et al. 2016) in R software, version 4.2.3 (R Core 2023), was employed to produce the rarefaction curves.

## Data resources

A total of 29,247 samples, covering 28,853 Angiosperms, 308 Pteridophytes, 56 Algae and 30 Bryophytes were collected in the study area. Of this total, 24,021 samples (82.13%) were known at species level, 3,713 (12.69%) at genus level, 1,148 (3.92%) at family level and only 366 (1.25%) remained unidentified. Some taxa were known at subspecies and variety level. The set of all the records collected for the present work was included in the dataset of occurrences of green plants, a water infrastructure project within the Caatinga Phytogeographical Domain, that are stored in the Herbário do Vale do São Francisco - HVASF (Siqueira-Filho 2025) and the Herbário de Referência do Sertão Nordestino - HRSN (Pifano 2025), both from the UNIVASF, located in Petrolina, Pernambuco State, Brazil. Duplicate plant samples were often donated to other Brazilian herbaria.

## Checklists

### Green plants (Viridiplantae) in 56,144 km² within the Caatinga Phytogeographic Domain, Brazil

#### Plantae s. s.

D25411C1-068F-51F7-9EC3-4DC3D13E655D

##### Notes

The checklist compiled 1,610 species, including 1,552 Angiosperms, 36 Pteridophytes, 13 Bryophytes and nine Algae. The complete list of species and their respective vouchers is in the Suppl. material 1.

## Analysis

### Richness, floristic composition and distribution of taxa

The Angiosperm species belong to 135 families and 683 genera. The greatest species richness in the checklist belongs to the families Fabaceae (246), Poaceae (89), Euphorbiaceae (75) and Malvaceae (71) and the genera *Cyperus* (with 27 species), *Croton* (24) and *Chamaecrista* (22). Notably, 39 families (28.88% of the total) and 412 genera of Angiosperms (60.32%) were represented by only one species.

**Figure 3.**
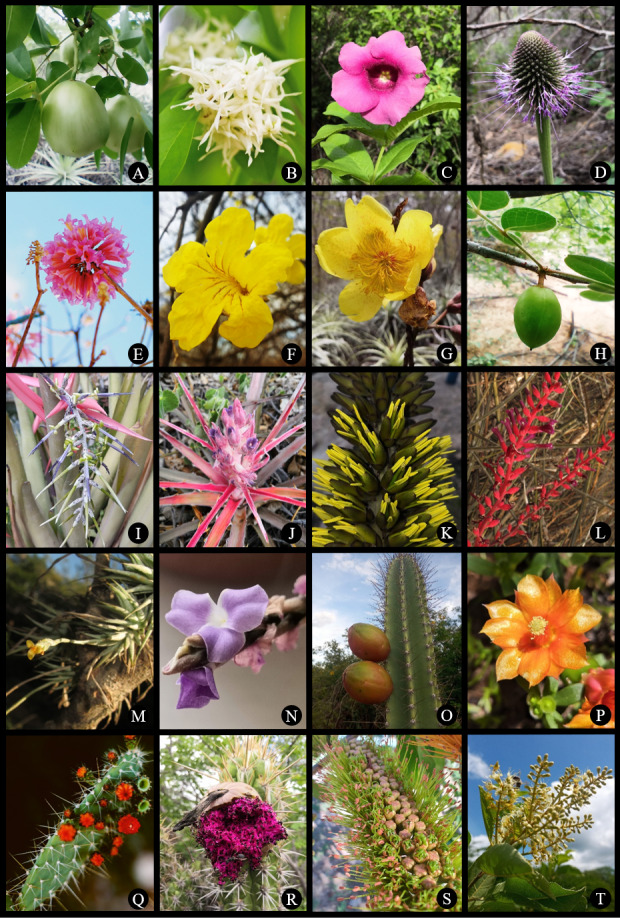
Species recorded in the Phytogeographic Domain of the Caatinga, along the sampling grid of the PISF. **A** *Spondias tuberosa*; **B** *Aspidosperma pyrifolium*; **C** *Allamanda blanchetii*; **D** *Chresta pacourinoides*; **E** *Handroanthus impetiginosus*; **F** *Handroanthus spongiosus*; **G** *Cochlospermum vitifolium*; **H** *Commiphora leptophloeos*; **I** *Billbergia porteana*; **J** *Bromelia arenaria*; **K** *Dyckia spectabilis*; **L** *Neoglaziovia variegata*; **M** *Tillandsia loliacea*; **N** *Tillandsia streptocarpa*; **O** *Cereus jamacaru*; **P** *Tacinga inamoena*; **Q** *Tacinga palmadora*; **R** *Xiquexique gounellei*; **S** *Combretum lanceolatum*; **T** *Combretum leprosum*. Photo credits: by L.F. Lima (A, D, G, H-K, N-P, R-T), NEMA (B, E, L, Q), V.M. Cotarelli (C, F), A.P. Fontana (M).

**Figure 4.**
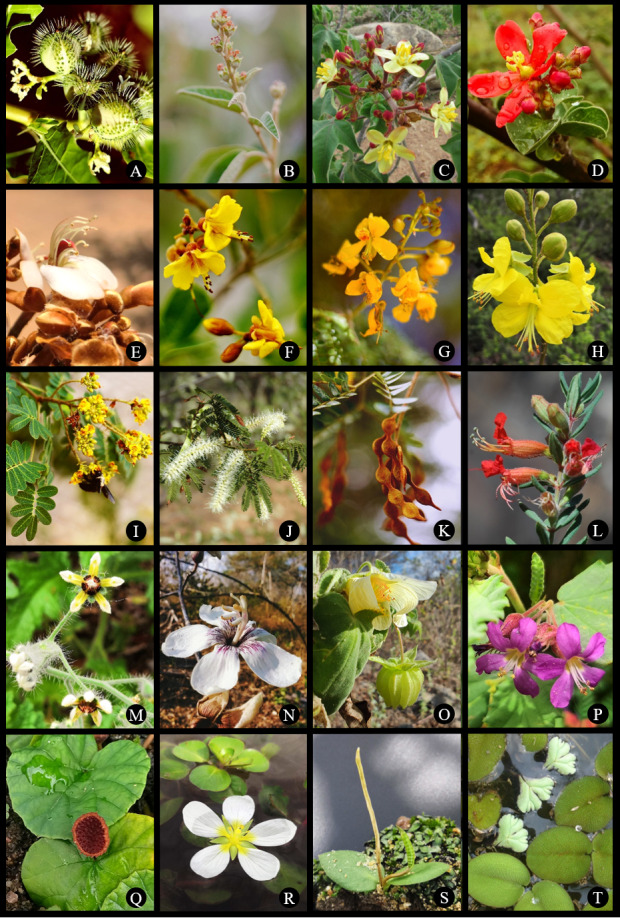
Species recorded in the Phytogeographic Domain of the Caatinga, along the sampling grid of the PISF. **A** *Cnidoscolus quercifolius*; **B** *Croton blanchetianus*; **C** *Jatropha mollissima*; **D** *Jatropha mutabilis*; **E** *Amburana cearensis*; **F** *Cenostigma pyramidale*; **G** *C. microphyllum*; **H** *Erythrostemon calycinus*; **I** *Libidibia ferrea*; **J** *Mimosa tenuiflora*; **K** *Pityrocarpa moniliformis*; **L** *Pleurophora pulchra*; **M** *Aosa rupestris*; **N** *Ceiba glaziovii*; **O** *Herissantia tiubae*; **P** *Melochia tomentosa*; **Q** *Dorstenia asaroides*; **R** *Ludwigia helminthorrhiza*; **S** *Ophioglossum nudicaule*; **T** *Ricciocarpos natans* and *Salvinia auriculata*. Photo credits: by NEMA (A-D, F, G, I, K), L.F. Lima (E, H, M-P, R, S), V.M. Cotarelli (J, L, Q, T).

In the Pteridophyte group, the checklist includes 13 families and 23 genera. The highest species richness within this group was found in Pteridaceae (11 species), followed by Anemiaceae and Salviniaceae, each with five species. The genera with the greatest species richness in this group were *Anemia* (4 species), *Salvinia*, and *Selaginella*, each with three species. Seven Pteridophyte families (18.91% of the total) and 17 genera (72%) were represented by a single species. For the Bryophyte group, the checklist includes 10 families, with Ricciaceae and Corsiniaceae exhibiting the highest species richness, comprising three and two species, respectively. The genera with the greatest species richness in this group were *Ricciocarpos* and *Cronisia*, each represented by two species (Suppl. material 1). Algae were represented in our checklist by the family Charophyceae, with eight species belonging to Chara, in addition to *Nitella cernua* A. Braun. Images of some of the species recorded in the study area can be found in Figs 3, 4.

None of the families, genera or species in the checklist was recorded in all grid cells of the study area. The family Fabaceae was the most widely distributed, with representatives present in 99% of the grid cells, followed by Euphorbiaceae (95.68%) and Malvaceae (92.24%). The genera *Croton*, *Tillandsia*, and *Senna* were found in 82.75%, 81.89% and 78.44% of the grid cells, respectively. *Tillandsia loliacea* was recorded in 68.96% of the grid cells, followed by *Cenostigma pyramidale* (67.24%) and Anadenanthera colubrina var. cebil (Griseb.) Altschul (60.34%). Species from 27 families and 146 genera were recorded in only one grid cell.

### Species classifications - Life forms, origin, endemism and threat status

A significant percentage of Angiosperm and Pteridophyte species in the checklist (40.68%) exhibit herbaceous life forms, followed by shrubs (24.47%), trees (13.29%), lianas/climbers (12.48%), subshrubs (7.45%) and aquatic-benthic, thalloid, tuft and palm forms (1.61%). Algal species predominantly exhibit an aquatic-benthic life form, while Bryophyte species are typically thalloid. The highest species richness amongst herbaceous plants was found in Poaceae and Cyperaceae. Fabaceae exhibited the greatest species richness across the following life-form categories: herbaceous, subshrub, shrub, tree and liana/climber. The palm life form was exclusively represented by species of Arecaceae. Amongst liana/climber plants, Convolvulaceae exhibited the highest species richness, particularly the genera *Ipomoea* and *Jacquemontia* (Suppl. material 1).

Most species in the checklist (91.67%) are native to Brazil, while a smaller proportion are naturalised (6.45%) or cultivated (1.86%). The families with the highest number of naturalised species were Poaceae (30 species), Fabaceae (12), Asteraceae (8) and Solanaceae (7), while Fabaceae (4), Poaceae, Cucurbitaceae and Annonaceae (3 each) had the highest number of cultivated species. The naturalised species *Lantana camara* L., *Eragrostis tenella* (L.) P. Beauv. ex Roem. & Schult. and *Eragrostis cilianensis* (All.) Vignolo ex Janch. were most collected in the sudy area, with 132, 95 and 82 samples, respectively. Amongst cultivated species, *Tamarindus indica* L., *Andropogon gayanus* Kunth and *Luffa cylindrica* Roem. were the most collected, collectively accounting for 38.46% of the total samples in this category.

The checklist includes 173 species and ten genera (*Alvimiantha*, *Ameroglossum*, *Anamaria*, *Dizygostemon*, *Fraunhofera*, *Harpochilus*, *Hydrothrix*, *Neesiochloa*, *Neoglaziovia* and *Telmatophila*) that are native to Brazil and endemic to the CPD. The families Fabaceae (34 species), Euphorbiaceae (16), and Cactaceae (11) had the highest number of species endemic to the CPD (Suppl. material 1). The checklist also includes 16 species listed in the Official National List of Endangered Flora Species (MMA 2022), with one classified as Critically Endangered (CR), ten Endangered (EN) and five Vulnerable (VU). Additionally, four of these species were represented by only one sample in the study area.

### Contribution to taxonomic and biogeographic shortfalls

Field expeditions of the PISF recorded the first occurrence of 284 species for one or more States in northeast Brazil or for the CPD. We highlight the first occurrences of 110 species for the States of Paraíba, Pernambuco (104), Ceará (82), Rio Grande do Norte (28) and Alagoas (15), in addition to 22 species recorded for the first time in the CPD (Suppl. material 1). The distribution of 13 non-native species in Brazil (naturalised or cultivated) is expanding to one or more States in the northeast region (Suppl. material 1). The geographic distribution of 28 endemic species from the CPD was expanded to one or more States in northeast Brazil by the PISF (Suppl. material 1). Additionally, the first occurrence of *Leptohyptis pinheiroi* in the CPD was recorded, a species classified as endangered in Pernambuco State (Suppl. material 1).

Two new species were discovered and described, based on plant samples collected by the PISF: *Pleurophora pulchra* J.A.Siqueira, Cotarelli, J.F.B.Pastore & T.B.Cavalc. (Siqueira-Filho et al. 2015, Fig. 4) and *Orthophytum alagoanum* Leme & Fontana (Leme et al. 2020). Furthermore, the plant samples donated by the PISF to different herbaria in Brazil contributed to the description of five other new species: *Amorimia pellegrinii* Almeida (Almeida et al. 2016), *Croton sertanejus* Sodré & Silva (Sodré and Silva 2022), *Hymenaea cangaceira* Pinto, Mansano & Azevedo (Pinto et al. 2017), *Erythroxylum pyan* Costa-Lima (Costa-Lima and Chagas 2018) and *Solanum caatingae* Knapp & Särkinen (Knapp and Särkinen 2018). These donated samples were cited as paratypes in the examined material of the new species.

**Figure 5.**
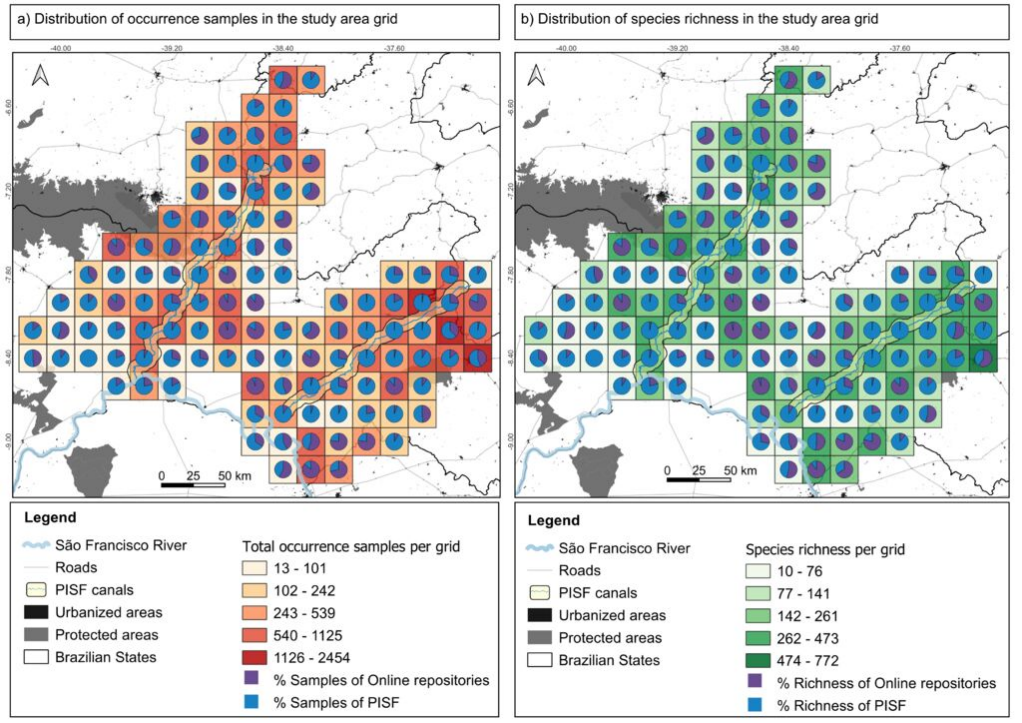
Distribution of occurrence records and green plant species richness in the Caatinga Phytogeographic Domain. **A** Total number of occurrence samples per grid cell in the study area; **B** Total number of species recorded per grid cell in the study area. The pie charts within each figure show the proportions of green plant occurrence samples (**A**) or species richness (**B**) per grid cell in the study area, as recorded either by the PISF floristic inventory or by online databases.

There are 43,775 plant records in the study area, combining the PISF dataset (29,247) with records from online repositories (14,528). Most of the cells in the study area (80.17%) had more plant samples collected by the PISF than those accessed through online repositories (Fig. 5; Suppl. material 2). Additionally, 38 cells in the study area had 90% or more of their plant samples obtained by the PISF (Fig. 5; Suppl. material 2).

**Figure 6.**
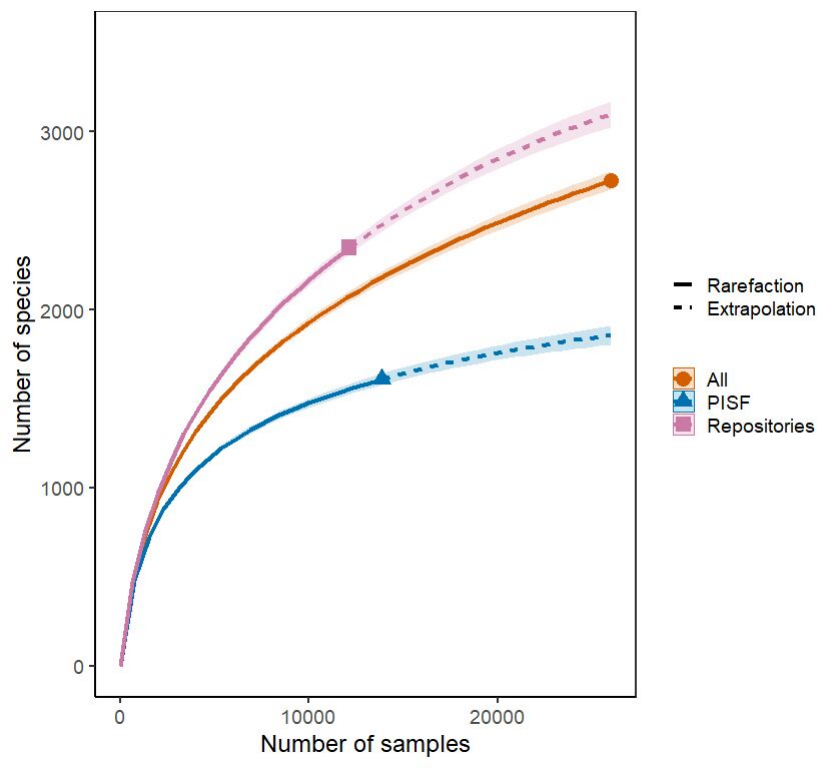
Rarefaction curves showing the growth trend of the accumulated number of species of green plants with increasing number of samples in the study area, utilising data from online repositories or data from field expeditions of the PISF or even these two unified datasets.

A total of 2,728 plant species were recorded in the study area, of which 1,223 species are common to all databases and 377 are exclusive to the PISF dataset. This expanded the known species richness of this area by approximately 14%. The PISF dataset accounted for more than 50% of the species richness within 82 grid cells of the study area (70.68% of the total cells) and more than 90% in another 29 grid cells (25%). The rarefaction curve computed with all datasets (PISF plus online repositories) shows no trend towards stabilisation of species richness with increased sampling (Fig. 6; Suppl. material 2). However, the rarefaction curve computed with the PISF dataset alone indicated a trend towards stabilisation in species numbers (Fig. 6; Suppl. material 2).

## Discussion

### Richness, floristic composition, distribution of taxa and species classifications

Pteridophytes, Algae and Bryophytes exhibit a significantly lower richness than Angiosperms in the Neotropical Ecozone (Murphy et al. 2019). Usually, Pteridophytes, Algae and Bryophytes occur in habitats with specific environmental conditions, such as higher humidity and lower air temperatures (Judd et al. 2009, Lee 2012). These conditions, however, are not permanent in most regions of the CPD. Moreover, they are generally neglected groups in plant sampling expeditions (Moura Júnior and Cotarelli 2019). That accounts for the low number of species of Pteridophytes, Algae and Bryophytes recorded in our study area compared to the number of Angiosperm species. Nonetheless, it is crucial to highlight that the richness of Pteridophytes in our checklist represents 63.63% of that known for the CPD (Flora e Funga do Brasil 2024), with 12 indications of new occurrences for this Domain or for one or more States in northeast Brazil. For Bryophytes, it represented 10% of the known richness for the CPD (Flora e Funga do Brasil 2024), with four indications of new occurrences for the Domain.

Fabaceae, Euphorbiaceae, Poaceae, Malvaceae and Asteraceae are amongst the families richest in plant species in checklists developed within the CPD (Machado et al. 2012, Costa et al. 2015, Lima et al. 2019, Lopes-Silva et al. 2019). The predominance of these families in the plant diversity of the CPD is attributable to the existence of 'oligarchies' of these taxa, resulting from the adaptations of their representatives to the distinct environmental requirements of the Domain (Moro et al. 2014, Fernandes et al. 2020). It is worth mentioning that Fabaceae encompasses a high number of cosmopolitan genera, as well as species that exhibit different life forms, reproduction strategies and dispersal (Souza and Lorenzi 2019). That may explain the high representation of Fabaceae in the species richness of herbaceous subshrub, shrub, tree and liana/climber life forms in our checklist. The plant checklists from the CPD (Moro et al. 2014, Fernandes et al. 2020) have also noted the representativeness of Fabaceae in the species richness of different life forms. Myrtaceae is not listed amongst the five families with the highest species richness in the checklists from the CPD (Moro et al. 2014, Fernandes et al. 2020), although Queiroz et al. (2017) cite this family as exhibiting high richness in arboreal plants, especially in areas of sedimentary Caatinga.

*Cyperus* has a high representation in the species richness of floristic surveys within Seasonally Dry Tropical Forests of the Neotropics (Linares-Palomino et al. 2015, Fernandes et al. 2020). This genus holds the second-highest number of species amongst the Cyperaceae family in Brazil, boasting 122 species, with 58 registered in the CPD (Alves et al. 2009, Flora e Funga do Brasil 2024). *Cyperus* species have effective propagation and dispersal strategies, enabling them to colonise and thrive in aquatic, terrestrial and ecotonal zones (Moura Júnior and Cotarelli 2019). Therefore, the high species richness of Cyperus in this checklist can be explained by two main factors: the widespread distribution of this genus throughout Brazil and the repeated field expeditions conducted by PISF, which involved sampling various types of aquatic and terrestrial ecosystems within the CPD. The recent synonymising of *Pycreus*, *Kyllinga* and *Oxycaryum* species into Cyperus may also have contributed to the high number of species in this latter genus in this checklist.

*Tillandsia* is rarely mentioned in floristic studies conducted in CPD patches (Costa et al. 2015, Cordeiro et al. 2018, Lima et al. 2019), despite this genus being amongst the richest in plant species within the Seasonally Dry Tropical Forests (Linares-Palomino et al. 2015, Fernandes et al. 2020). When we consider that the species richness of *Tillandsia* in this checklist is 500% higher than that recorded in the online databases (SpeciesLink, Reflora and GBIF) within the study area, it becomes evident that this genus is neglected in the flora surveys conducted within CPD.

The high species richness of herbaceous life forms has been highlighted in plant species surveys conducted in different areas of the CPD (Machado et al. 2012, Costa et al. 2015, Moro et al. 2016, Lima et al. 2019), as evidenced in this checklist. Nevertheless, knowledge about the taxonomic diversity of non-woody plants in the CPD remains limited compared to that obtained for trees or shrubs (Araújo 2014, Santos and Figueiredo 2018) or even life forms associated with aquatic plants, such as thalloid or benthic-aquatic (Moura Júnior and Cotarelli 2019). It was expected that Poaceae and Cyperaceae would stand out in species richness and number of samples amongst herbaceous plants, since these families were cited as the most representative in the taxonomic diversity of herbaceous plants in the CPD (Fernandes et al. 2020).

According to Zenni (2015), the CPD harbours approximately 30% of Brazil’s 525 non-native vascular plant species. Furthermore, data from Flora e Funga do Brasil (2024) indicate that 195 naturalised and 32 cultivated species have been recorded in the CPD amongst angiosperms, algae, ferns and lycophytes. In this context, the richness of naturalised and cultivated species in this checklist represents a significant sample of the CPD’s non-native flora. Poaceae and Fabaceae exhibit high naturalised species richness across all phytogeographic domains of Brazil (Zenni 2013, Zenni 2015). The adaptation of a naturalised species may advance to the stage of the biological invasion when the species disperses further and establishes highly dense, self-sustaining populations in new areas (Richardson et al. 2001). Therefore, the non-native species in our checklist that have shown an expansion in their distribution may serve as an early warning of potential invasions. We thus encourage botanists and ecologists to assess the dispersal potential and population density of these species within the CPD.

The endemic species richness recorded in our checklist contradicts the assertion that regions of Seasonally Dry Tropical Forests host a low number of endemic plant species (Leal et al. 2003). The number of endemic species in our checklist corresponds to 15% or 32% of the CPD’s endemic plant richness, based on data from Flora e Funga do Brasil (2024) and the checklist compiled by Fernandes et al. (2020), respectively. However, 23 endemic species in our checklist were represented by a single specimen in the study area, suggesting that their populations may be rare in this region and/or that the knowledge of their distribution remains incomplete. The low number of endangered species recorded in our study area is unsurprising. Given the increasing number of degraded or lost areas in the CPD over the past 30 years (Albuquerque et al. 2012) and the climate change scenarios projected for the end of this century, a large proportion of the CPD’s endemic plant species — particularly those already threatened with extinction — are at high risk of disappearing within the next 80 years (Moura et al. 2023). In 2013, most of the remaining CPD fragments were smaller than 10,000 hectares (Leal et al. 2003) and their size has continued to decline over the past decade. The CPD harbours the highest number of plant genera at risk of extinction amongst all Seasonally Dry Tropical Forest regions worldwide (Fernandes and Queiroz 2018).

### Environmental licensing addressing biodiversity shortfalls

The rarefaction curve computed using primary data revealed that the PISF field expeditions significantly reduced plant biodiversity shortfalls (Linnaean and Wallacean) within the study area. However, the rarefaction curve generated from all collected data (primary and secondary) suggests that significant efforts are still needed to address plant biodiversity gaps in this region.

Although systematic field efforts in an area corresponding to 10% of the CPD provided access to 47% of the plant richness in this domain (*sensu* Fernandes et al. (2020)), these efforts would need to be expanded to encompass all plant species present in the study area. The plant checklists of the CPD were compiled from published studies and online data repositories (Moro et al. 2014, Fernandes et al. 2020) had already highlighted the need to expand collection efforts across different habitats within the CPD to reduce knowledge gaps regarding plant taxonomic diversity in this domain.

A significant portion of the knowledge on CPD taxonomic diversity is derived from floristic and phytosociological studies, based on specific or local inventories (Moro et al. 2014), predominantly conducted in protected areas or near urban centres, in easily accessible locations (Oliveira et al. 2016). Most of these studies are of short to medium duration and are primarily part of undergraduate, Master's or Doctoral research projects. Additionally, most studies are funded by Brazilian government agencies dedicated to supporting scientific research (e.g. CNPq and CAPES). Floristic and phytosociological studies in the CPD that sample extensive areas through successive field expeditions within the same study area are scarce (Siqueira-Filho 2012, Costa et al. 2015, Vitório et al. 2019). Funding for this green plant checklist was provided through the environmental licensing of a water infrastructure project, an uncommon funding source for biodiversity data collection studies within Brazil's phytogeographic domains. The extended duration of data collection in the checklist is also atypical of most studies addressing plant taxonomic diversity in these domains.

The description of new species and the first records of hundreds of species for the CPD or for one or more States in northeast Brazil underscore the importance of this checklist in addressing the first and second biodiversity shortfalls (Linnaean and Wallacean, respectively). These shortfalls necessarily influence all other gaps in biodiversity knowledge, as they hinder the understanding of species ecology and evolution (Hortal et al. 2015). The absence of empirical data on the characteristics of unknown species or the geographic distribution of known species affects our comprehension of their adaptation to abiotic conditions (Hutchinsonian shortfall). It also impacts our understanding of how ecological and functional traits vary across time and space and how they compare to those of other species (Raunkiaeran shortfall) (Hortal et al. 2015). Therefore, checklists conducted prior to new infrastructure developments are valuable for expanding knowledge of the ecological processes shaping plant communities (Moura et al. 2018). A portion of the floristic data obtained through the environmental licensing of the PISF, for instance, has been used to estimate plant abundance (Oliveira et al. 2020), taxonomic diversity (Simpson's index) and functional diversity of plants in CPD areas (Oliveira et al. 2022), as well as to assess the distribution of invasive plant species in these areas (Asth et al. 2021), including their implications for biodiversity conservation (Oliveira et al. 2020).

However, to address biodiversity shortfalls and enable informed decision-making in biodiversity conservation, careful planning of environmental licensing studies is essential (Oliveira et al. 2020). The PISF environmental plan, for example, had three main objectives related to the flora inventory. First, to ensure that the flora of the entire direct or indirect influence area of the project were uniformly sampled, with collections conducted across different habitats within each grid cell and re-sampling carried out over several years and across multiple months. Second, to include all terrestrial and aquatic life forms of green plants, allowing access to taxonomic or ecological groups that are poorly studied in semi-arid regions (e.g. aquatic macrophytes, mosses, liverworts, hornworts, ferns and lycophytes). Third, to ensure accurate taxonomic identification of all plant species recorded in the study area. Achieving this required dedicated curation of specimens in the herbarium, exchange of exsiccatae with other botanical collections, collaboration with numerous specialists and the availability of data and collected specimens.

Species surveys covering large geographic areas prevent partial or erroneous interpretations of species distribution, thereby reducing the likelihood of false indications of rarity, endemism or extinction risk (Whittaker and Fernández-Palacios 2007). However, implementing and sustaining multiple species surveys at large spatial scales over extended periods presents significant challenges. When the objective is to describe species richness and composition at a landscape scale, the 'single large or several small' (SLOSS) debate centres on two species survey strategies: first, a single, geographically extensive, long-term survey; second, multiple localised surveys at smaller scales (Yaynemsa 2023). This debate is often linked to the conservation biology paradigm "single large or several small", based on island biogeography theory. This paradigm asks if a single large protected area is a more effective strategy for biodiversity conservation than several small reserves within fragmented habitats.

In summary, our primary data have resulted in an increase of species richness and plant samples in CPD areas. We have distributed duplicates of specimens to several herbaria across Brazil, facilitating access for taxonomists specialising in specific orders, classes, families and genera of plants. As a result, numerous taxa have been identified at the species level, expanding our understanding of the geographical distribution of hundreds of species in the CPD and contributing to the discovery of new species. It is highly likely that specimens collected through the PISF will, in the future, contribute to the description of additional plant taxa. However, it is necessary to enhance field efforts to minimise knowledge gaps regarding the taxonomic diversity of algae, bryophytes and pteridophytes in the CPD. Likewise, we emphasise the importance for taxonomic botanists to include species from all life forms in their floristic surveys conducted in CPD areas, rather than focusing solely on trees or woody flora.

We are confident that all the scientific advancements presented in this paper were made possible through consistent financial support for field expeditions, payment of professionals and maintenance of the HRSN and HVASF herbaria infrastructure over the years. As mentioned throughout this manuscript, all logistical, material and equipment costs associated with this plant species survey were financed through the environmental licensing of infrastructure work. In Brazil, this is uncommon. Most biodiversity studies in the country are funded by government agencies that support scientific research (e.g. CNPq and CAPES), which primarily finance short- and medium-term research at relatively small spatial scales (CNPq 2023, CAPES 2024). In contrast, environmental programmes associated with environmental licensing in Brazil require long-term studies, including repeated surveys of areas affected by infrastructure works, in order to mitigate the socio-environmental impacts of these ventures (Moura et al. 2018). Consequently, these environmental programmes aimed at biodiversity conservation always involve multidisciplinary teams of professionals with extensive expertise in species identification and long-standing field experience.

Finally, we stress that environmental licensing, supported by appropriate legislation, is fundamental to ensuring a minimum balance in the sustainability tripod (economic, social and environmental). When environmental licensing is weakened or dismantled, everyone loses, especially biodiversity. Therefore, continuous improvement and strengthening of environmental legislation in Brazil are necessary, not the opposite.

## Supplementary Material

XML Treatment for Plantae s. s.

4C852ED5-879E-5889-8AC0-B448B71F069A 10.3897/BDJ.13.e159621.suppl1 Supplementary material 1 Table S1. Data type species data Brief description List of groups (Algae, Angiosperms, Bryophytes, Ferns & Lycophytes), families, genera and species collected in floristic inventories, conducted in areas of the Caatinga Phytogeographic Domain (CPD), through actions linked to the environmental licensing of the São Francisco River Integration Project with Hydrographic Basins of the Northern Northeast (PISF), in addition to data on endemism to CPD, threat status (by Ordinance 300/2022 of the Ministério do Meio Ambiente do Brasil – MMA), geographic origin (Naturalised non-native to Brazil; Cultivated non-native to Brazil; or Native to Brazil), life form and new occurrences of the species (from the State of northeast Brazil or CPD). Legend: NA - Not applicable. File: oo_1397523.xlsx https://binary.pensoft.net/file/1397523 Vinicius Messas Cotarelli, Edson Gomes de Moura-Júnior, Liliane Ferreira Lima, André Paviotti Fontana, Lorenna Campos Cruz, Renato Garcia Rodrigues, Daniel Salgado Pifano

7678D66E-B1AE-586C-8AFC-23BA310DAF8E 10.3897/BDJ.13.e159621.suppl2 Supplementary material 2 Table S2 Data type plant samples, richness Brief description Database on samples, richness and checklist found in the grid cells of the sampling grid of the studied area, showing the sampling effort of the PISF and the online repositories within the Caatinga Phytogeographic Domain (CPD). File: oo_1397573.xlsx https://binary.pensoft.net/file/1397573 Vinicius Messas Cotarelli, Edson Gomes de Moura-Júnior, Liliane Ferreira Lima, André Paviotti Fontana, Lorenna Campos Cruz, Renato Garcia Rodrigues, Daniel Salgado Pifano
